# Harnessing Natural Product Compounds to Target Dormancy Survival Regulator (DosR) in Latent Tuberculosis Infection (LTBI): An In Silico Strategy Against Dormancy

**DOI:** 10.3390/arm93030019

**Published:** 2025-06-16

**Authors:** Mandeep Chouhan, Mukesh Kumar, Vivek Dhar Dwivedi, Vivek Kumar Kashyap, Himanshu Narayan Singh, Sanjay Kumar

**Affiliations:** 1 Biological and Bio-Computational Lab, Department of Life Science, School of Basic Sciences and Research, Sharda University, Greater Noida 201310, UP, India; 2 Department of Biophysics, All India Institute of Medical Sciences, New Delhi 110029, India; 3 Bioinformatics Research Division, Quanta Calculus, Greater Noida 201310, UP, India; 4 Division of Cancer Immunology and Microbiology, Medicine and Oncology Integrated Service Unit, School of Medicine, University of Texas Rio Grande Valley, McAllen, TX 78504, USA; 5 South Texas Center of Excellence in Cancer Research, School of Medicine, University of Texas Rio Grande Valley, McAllen, TX 78504, USA; 6 Department of Systems Biology, Columbia University Irving Medical Center, New York, NY 10032, USA; 7 Department of Radiology, Memorial Sloan Kettering Cancer Center, New York, NY 10065, USA; 8 DST-FIST Facility, Sharda University, Greater Noida 201310, UP, India; 9 Centre of Excellence for Artificial Intelligence in Medicine, Imaging & Forensics, Sharda University, Greater Noida 201310, UP, India

**Keywords:** tuberculosis, dormancy, DosR, structure-based virtual screening, molecular dynamics simulation

## Abstract

**Highlights:**

**What are the main findings?**

- M3 among all screened natural product based compounds, demonstrated the strongest binding affinity and stability with DosR protein.
- M3 outperformed reference compound Ursolic acid in molecular docking, MD Simulation and MM/GBSA.

**What is the implication of the main finding?**

- M3 emerges as a potential candidate for further experimental studies against LTBI.
- Targeting DosR-regulated dormancy mechanisms could contribute to the development of more effective treatments against LTBI.

**Abstract:**

Dormancy occurs when *Mycobacterium tuberculosis* (Mtb) enters a non-replicating and metabolically inactive state in response to hostile environment. During this state, it is highly resistant to conventional antibiotics, which increase the urgency to develop new potential drugs against dormant bacilli. In view of this, the dormancy survival regulator (DosR) protein is thought to be an essential component that plays a key role in bacterial adaptation to dormancy during hypoxic conditions. Herein, the NP-lib database containing natural product compounds was screened virtually against the binding site of the DosR protein using the MTiopen screen web server. A series of computational analyses were performed, including redocking, intermolecular interaction analysis, and MDS, followed by binding free energy analysis. Through screening, 1000 natural product compounds were obtained with docking energy ranging from −8.5 to −4.1 kcal/mol. The top four lead compounds were then selected for further investigation. On comparative analysis of intermolecular interaction, dynamics simulation and MM/GBSA calculation revealed that M3 docked with the DosR protein (docking score = −8.1 kcal/mol, RMSD = ~7 Å and ΔG Bind = −53.51 kcal/mol) exhibited stronger stability than reference compound Ursolic acid (docking score = −6.2 kcal/mol, RMSD = ~13.5 Å and ΔG Bind = −44.51 kcal/mol). Hence, M3 is recommended for further validation through in vitro and in vivo studies against latent tuberculosis infection.

## 1. Introduction

The progression of tuberculosis (TB) infection typically depends on the dynamic interaction between *Mycobacterium tuberculosis* (Mtb) and the host’s immune system. Mostly, an effective immune response in infected individuals restricts proliferation of bacterium by isolating the bacteria within the granuloma, thereby halting the progression of the disease. However, the bacteria may remain dormant in untreated individuals within granulomas, if not eliminated, which leads to latent tuberculosis infection (LTBI) that can persist for a lifetime [1,2,3,4]. One-quarter of the global population is estimated to be infected with Mtb, which represents a massive reservoir of active TB cases. Approximately 5–10% of individuals with LTBI will develop active TB disease during their lifetime, with significantly higher risk in immunocompromised persons, like those infected with HIV or diabetes [5]. The ability of tubercle bacilli to remain dormant in a metabolically inactive form without causing any symptom makes this bacterium a remarkably active pathogen as it exists silently for decades in the host and remains asymptomatic [6].

Current LTBI treatment regimens include drugs such as isoniazid, rifapentine, and rifampin, which are often used alone or in combination for several months [7]. However, these drugs were developed for active TB and are much less effective against LTBI. Furthermore, these treatment regimens exhibit hepatotoxicity and prolong therapy durations [8]. The situation of TB infection has become more complicated with the rise in multidrug-resistant (MDR) TB and extensively drug-resistant (XDR) TB, against which the conventional treatment regimens have become ineffective [9]. Therefore, there is an emergency in which it is necessary to develop a new therapeutic agent that can effectively target dormant bacteria for the complete eradication of TB.

During latent infection, Mtb undergoes a physiological transition from an actively replicating form of bacteria to a non-replicating, latent form [10,11]. This shift is primarily mediated by the two-component regulatory system, which responds to environmental stressors such as hypoxia, nutrition depletion, elevated nitric oxide levels, carbon monoxide, vitamin C, and acidic pH within granulomas [12,13,14,15,16]. Typically, a two-component regulatory system comprises a membrane-bound sensor kinase and a cytosolic response regulator. Sensor kinase senses the external signals, and the cytosolic response regulator regulates the cellular responses. Due to these essential properties, these systems are targeted to develop potential mycobacterial drugs. The genome of Mtb encodes ~190 regulatory proteins and 11 two-component systems. Out of these 11 two-component systems, DosR-DosS-DosT (DevR-DevS-Rv2027c) is the most widely studied. It consists of two sensor kinases and one response regulator, i.e., (DosS, DosT) and (DosR), respectively [6,17].

The DosR system activates a regulon which contains approximately 48 genes, and is generally referred to as DosR regulon [4,18,19,20]. DosR-regulated genes encode the proteins involved in various cellular processes, including nitrogen metabolism, immune evasion, redox homeostasis, and cell wall synthesis. Although, the specific role of DosR regulon genes in the dormancy of bacteria is not much explored. An in-depth understanding of their functional role is crucial in order to understand their involvement in the long-term survival and persistence of bacteria [21]. Additionally, several antigens, such as Rv2029c, Rv2628, Rv2627c, and Rv1733c, produce significant interferon-γ (IFN-γ) responses in positive tuberculosis skin test (TST+) individuals [22]. However, the pathophysiological functions of these proteins are not clear. Further investigation should be conducted to understand the mechanisms by which the bacterium employs its latency-associated antigens to alter host immune responses [23].

**Figure 1:**
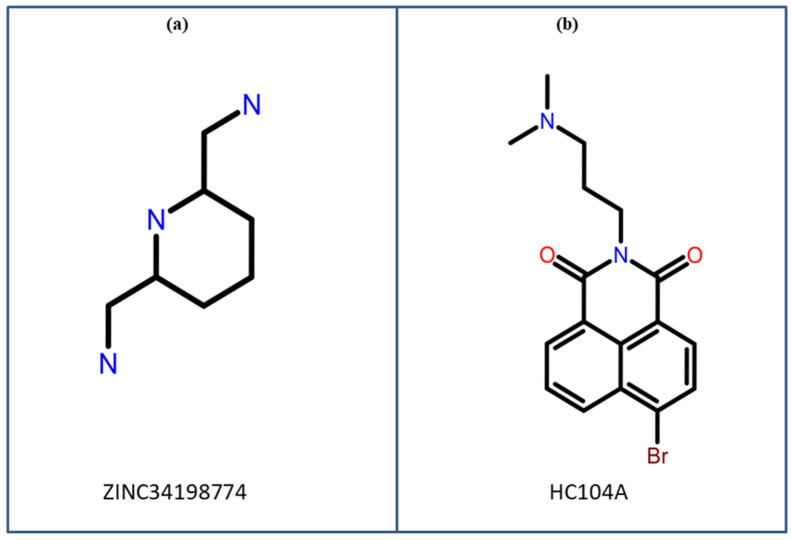
Structures of previously identified anti-tuberculosis compounds (**a**) ZINC34198774 and (**b**) HC104A, that showed structural similarity with the identified hits (M1–M4).

Given the limitations of current treatment regimens and the threat posed by LTBI, targeting the DosR-DosS-DosT system by inhibiting the DosR protein offers a strategic approach for LTBI infection inhibition. In view of this, targeting bacterial dormancy pathways present a promising strategy to enhance the efficacy of existing TB treatments and reduce the global disease burden because current treatment regimens of TB is most effective on actively replicating bacteria, like rifampicin, isoniazid, ethambutol, and pyrazinamide [24,25]. According to Gupta et al. (2009), the binding of the DosR protein to its cognate DNA can be blocked using 2-chloro-N-[4-[[4-(2-ketochromen-3-yl)benzoyl]amino]-2-methoxy-phenyl]benzamide, through blocking the inactive state of this protein [2]. Shanmuga Priya et al. (2022) found ZINC34198774 as a possible compound that targets DosR regulon, specially Rv2004c gene (a gene linked to rough morphology and virulent strain) [26]. Moreover, Jee et al. (2023) utilized computational methods in their study to explore natural metabolite Ursolic acid to identify it as an inhibitor of DosR protein [6]. Likewise, Sharma et al. (2023) also used computational techniques to investigate natural compounds against dormancy of mycobacteria, and they identified icariin as a potential candidate [27]. Figure 1 shows 2D structures of previously identified compounds that can potentially inhibit DosR protein which are structurally similar to our top candidates (M1–M4), supporting the rationale behind our virtual screening strategy.

In current study, our aim is to identify a natural-product-based compound against the DosR protein for latent TB infection. Here, DosR has been investigated using virtual screening and evaluation of compounds through molecular docking, MDS, and binding free energy calculations to identify a natural-product-based potential compound that could disrupt DosR functions under hypoxic conditions to overcome latent TB infection. Furthermore, previously identified compound Ursolic acid has been used as a reference molecule for comparison study. Our aim of this study was to identify a potential compound which is capable of disrupting DosR-regulated dormancy that can serve as a lead candidate for anti-LTBI drug development in future.

## 2. Methods

### 2.1. Retrieval and Preparation of Protein

The 3D structure of the DosR protein of Mtb with PDB ID: 3C3W, homo-dimer (chain A and B), with resolution of 2.20 Å [28], was retrieved from the RCSB protein data bank [29] (https://www.rcsb.org/) (accessed on 30 August 2024). The structure of the DosR protein was processed by Dock Prep tool in UCSF Chimera 1.15 using default settings [30,31]. The steps followed the deletion of chain B, water molecules, solvent, ions, and non-polar hydrogen atoms, then assignment of Gasteiger charges and addition of polar H-atoms to the target protein. These polar H-atoms are essential for the formation of H-bonding, and Gasteiger charges are necessary for the ionic interactions between the ligands and protein.

### 2.2. Selection of Compound Library

The NP-lib database (4 September 2024) from MTiOpenScreen web server was used for virtual screening, as it contains the purchasable natural product compounds collection. This database was built using a filtering protocol based on physiochemical and toxicophore properties. In addition, a visual inspection was carried out to eliminate the compounds, which are not suitable for virtual screening and molecular docking, as well as an assessment of the purchasability according to the ZINC15 database. The NP-lib database contains 1228 stereoisomers of natural products, which correspond to 653 single isomers [32]. Moreover, Ursolic acid with PubChem CID: 64945 was a previously identified inhibitor for DosR protein [6], and is used as reference compound in the current study for comparative analysis against screened compounds.

### 2.3. Protein Active Site Residue Prediction

The CASTp (Computed Atlas of Surface Topography of proteins) server was used to predict the binding site residues (Val55, Leu57, Gly60, Ala61, Ile63, Thr82, Leu161, Leu165, Ala167, Leu182, Val185, Ser186, Leu189, Met194, Gly195, Ala197, and Thr198) in the DosR protein [6,33]. Particularly, residue Thr82 is a very crucial residue, which is involved in phosphorylation-mediated activation of the DosR protein, and its positioning influences cooperative DNA binding, which is essential for proper gene activation [34]. These residues were used to generate the grid box during structure-based virtual screening.

### 2.4. Structure-Based Virtual Screening

Structure-based virtual screening (SBVS) is a method which is used to screen the library of small compounds and pinpoint compounds that are most likely to bind to a specific target protein [25,35,36]. MTiOpen Screen identifies the potential compound by estimating the binding energy of the compound against the target protein. Additionally, three binding poses were generated for each compound [32]. Herein, SBVS was carried out against the DosR protein using the NP-lib database to identify the potential compound through the MTiOpenScreen web server [32]. The prepared protein was uploaded in PDB format, and the NP-lib database was selected as a compound library to the MTiOpenScreen web server. Furthermore, residues (Val55, Leu57, Gly60, Ala61, Ile63, Thr82, Leu161, Leu165, Ala167, Leu182, Val185, Ser186, Leu189, Met194, Gly195, Ala197, and Thr198) were used as binding site residues to generate a grid for the virtual screening process. This result was evaluated based on affinity score, and the highest ranked compounds were then selected to check their inhibitory potential against DosR, which plays a critical role in bacterial persistence.

### 2.5. Molecular Docking

**Figure 2:**
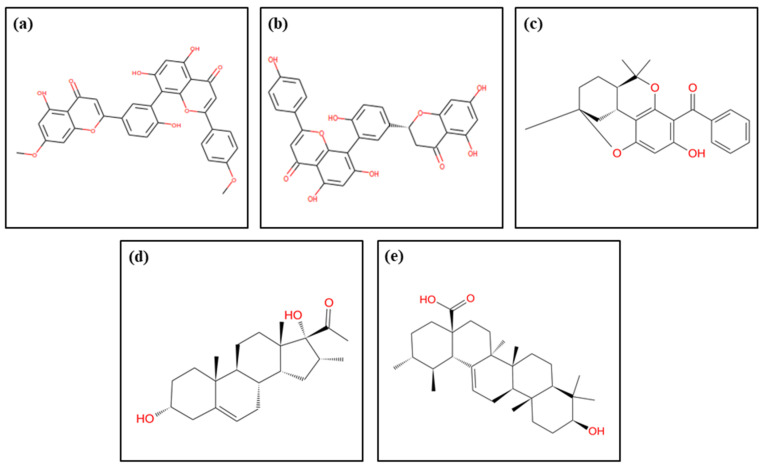
Structures of the selected natural product compounds: (**a**) M1, (**b**) M2, (**c**) M3, (**d**) M4, and (**e**) reference compound (Ursolic acid).

Following virtual screening, the top four compounds retrieved from the SBVS result were again redocked for further validation purpose (Figure 2). Redocking was carried out at the same binding pocket that covers all the binding site residues, which were used in the virtual screening process using the AutoDock Vina plugin setup in Chimera 1.15 using default parameters [30,35,36]. The grid box had dimensions of 19.42 × 31.95 × 29.44 Å at (X, Y, and Z) axes, while the center coordinates were 2.93 × 47.71 × 54.5 Å, which covered all the binding site residues inside it. Briefly, structure minimization was carried out for docked complexes of protein and screened drugs using the structure minimization tool in USCF Chimera-1.15 [31], and these complexes were saved in PDB format. Finally, the top poses were chosen for intermolecular interaction profiling based on the highest negative docking score and lowest RMSD (set value 0 by default), where the ligand–receptor interaction module was used in the academic version of Maestro 12.3 [37].

Here, different intermolecular interactions between active site residues of protein and compounds of docked complexes, i.e., H-bonds, hydrophobic, pi-cation, pi-pi interaction, negative residual interaction, positive residual interaction, polar interaction, glycine, and formation of salt bridges, were calculated under default conditions. Additionally, same criteria were used to dock reference compound i.e., Ursolic acid, with DosR protein. Both 2D and 3D images of docked complexes of selected compounds and reference compound were generated using Maestro 12.8 free version.

### 2.6. Molecular Dynamics Simulation

Molecular dynamics simulation (MDS) serves as a pivotal computational tool in structure-based drug design, enabling atomic-level investigation of biomolecular interactions and conformational dynamics [35]. It was performed to evaluate the stability and intermolecular interactions of the selected docked complexes over a 200 ns timescale using the Desmond module of Schrödinger Maestro [38,39]. The simulation system was prepared by solvating the docked complexes in an orthorhombic box (10 × 10 × 10 Å^3^) using the TIP3P explicit water model. To replicate physiological conditions, counter ions (Na^+^ or Cl^−^) were added to neutralize the system, and a 0.15 mol/L Na^+^Cl^−^ solution was introduced [40]. Positional restrictions were applied to the solute during the energy minimization process, which includes minimization through the Steepest Descent method, which is again followed by the Conjugate Gradient approach. The system was minimized for 2000 steps with a 1.0 kcal/mol/Å convergence threshold. NVT (constant number of particles, volume, and temperature) and NPT (constant number of particles, pressure, and temperature) ensembles and two phases of equilibration were performed using the Berendsen thermostat and barostat. The system exhibited relaxation time of 1 ps and 2 ps, after being equilibrated at a temperature of 300K and an atmospheric pressure of 1.013 bar, respectively. The Nosé-Hoover thermostat and the Martyna-Tobias-Klein barostat were used to control temperature and pressure throughout the whole production run.

Short-range interactions were calculated using 9 Å cutoff, whereas long-range electrostatic interactions were handled using the Particle Mesh Ewald (PME) method. The RESPA (Reversible Reference System Propagator Algorithm) integrator was applied for bonded interactions, using a 2 fs time step [41]. The OPLS3 force field was used to parameterize the system [42]. Simulation trajectories were analyzed using the Simulation Interaction Diagram (SID) module to assess root mean square deviation (RMSD), root mean square fluctuation (RMSF), and protein–ligand interaction profiles for all selected docked complexes.

### 2.7. Calculation of Molecular Mechanics/Generalized Born Surface Area (MM/GBSA)

The binding free energy of the selected complexes was calculated using the MM/GBSA method using the Prime module of Schrödinger Maestro [43]. This implicit solvent modeling approach is well known for accurately calculating binding energies from MDS trajectories.

In the current study, MM/GBSA calculation was performed using energy-stabilized frames from the last 20 ns of MDS. To calculate the net binding free energy, “thermal_mmgbsa” Python script was used. Key energy components, including ΔGBind Coulomb, ΔGBind covalent, ΔGBind H-bond, ΔGBind lipo, ΔGBind vdW, and ΔGBind Solv GB, were evaluated through MM/GBSA analysis, which was carried out using the VSGB 2.0 solvation model [44,45]. To ensure statistical reliability, the computed binding energy values were averaged, and their standard deviations were determined. The MM/GBSA method has been extensively validated and applied in computational studies, demonstrating its efficacy in predicting ligand–protein binding affinities.

The following standard equation was used to calculate the binding energy (MM/GBSA ΔGbind).

MM/GBSA ΔG_bind_ = G_complex_ − G_receptor_ − G_hit ligand_

where G_complex_ denotes total free energy of the protein–ligand complex; G_receptor_ denotes free energy of the unbound protein; G_hit ligand_ denotes free energy of the unbound ligand; and ΔG_bind_ predicts the binding affinity of the protein–ligand complexes and quantifies the stability of docked complexes.

## 3. Results

### 3.1. Structure-Based Virtual Screening (SBVS) and Redocking Analysis

The screening of the NP-lib database against the DosR protein produced 1000 compounds with docking score ranging from −8.5 to −4.1 kcal/mol (Supplementary Table S1). Furthermore, the top four lead compounds from this initial screening step were then redocked (Figure 2), revealing improved binding scores varying from −8.7 kcal/mol to −8.2 kcal/mol; they also occupied the binding site of DosR as mentioned in Table 1. Interestingly, all the lead compounds (ZINC000003594862 (M1), ZINC000059779788 (M2), ZINC000230017540 (M3), and ZINC000253501597 (M4)) exhibited a docking score greater than −8.2 kcal/mol in comparison to the reference compound Ursolic acid (−6.2 kcal/mol).

### 3.2. Intermolecular Interaction Analysis

**Figure 3:**
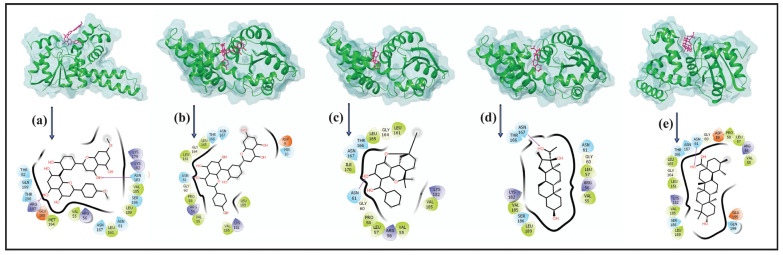
2D and 3D interaction profiles for the protein–ligand docked complexes; (**a**) DosR-M1, (**b**) DosR-M2, (**c**) DosR-M3, (**d**) DosR-M4, and (**e**) reference compound DosR-(Ursolic acid). Three-dimensional and 2D structures were generated from Maestro 12.8 version. In the 3D structure, the compound is represented by ball and stick model (pink color), and protein is represented by ribbon in constant green color. In 2D interaction diagram, hydrogen bonds (violet arrows), hydrophobic (green), positive (violet), negative (red), polar (blue), and glycine (creamy white) interactions are presented to be involved in docked complexes.

The intermolecular interactions of the top four compounds docked with the DosR protein of Mtb were analyzed to facilitate the formation of stable protein–ligand docked complexes. All four selected compounds demonstrated a significant position within the binding site of the DosR protein (Figure 3) and shared some common residues of the target protein for intermolecular interactions (Table 2, Figure 3).

The ability of compounds to inhibit DosR mainly depends on the number of hydrogen bonds they can form with the residues of the DosR protein. Herein, only compound M1, M3, and M4 exhibited hydrogen bond formation, whereas compound M2 showed no hydrogen bonding formation in the docked complexes. Notably, polar and hydrophobic interactions with critical residues of DosR were observed. M1 exhibited two instances of hydrogen bonding with residues Arg197 and Asn183; M4 displayed hydrogen bonding with Asn167; and the M3 and reference compound showed hydrogen bonding with residue Asn61. All selected compounds formed some similar interactions and showed a similar binding profile, including significant hydrophobic, polar, and other intermolecular contacts with residues, such as Val55, Asn167, Val185, Asn61, Thr166, Asn167 Arg56, Lys182, and Gly60. The reference compound, CID_64945, exhibited similar interactions to compound M3.

All the compounds depicted overlapping interaction profiles when comparing the interaction profiles of docked complexes. Furthermore, particular interactions were observed with residues Val55, Val185, Asn61, Asn167, Arg56, and Lys182, which are hypothesized to increase the stability of the complex; they might also interrupt the transcriptional regulation activity of the DosR protein. In comparison to compound M2 and the reference compound, docking analysis showed that the compounds M1, M3, and M4 may have the potential to inhibit the DosR protein through the formation of H-bonds and non-covalent interactions. Therefore, these compounds may have the ability to disrupt the dormancy response regulated by DosR, which is a crucial survival mechanism of Mtb under hypoxic conditions.

### 3.3. Molecular Dynamics Simulation Analysis

#### Structural Stability and Equilibration Dynamics

**Figure 4:**
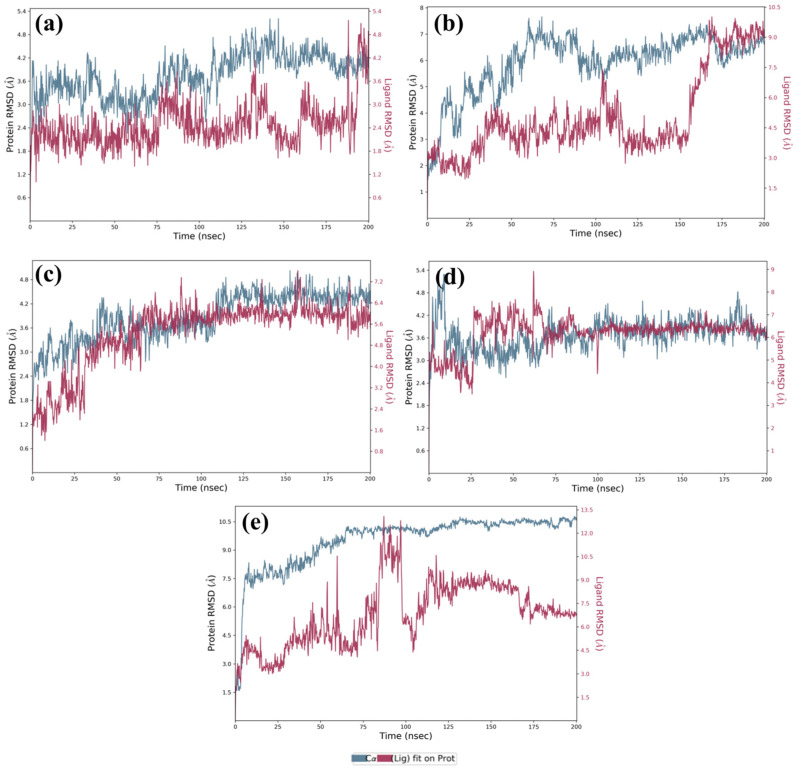
Root mean square deviation plot for the backbone atoms of DosR protein with selected natural product compounds: (**a**) DosR-M1, (**b**) DosR-M2, (**c**) DosR-M3, (**d**) DosR-M4, and (**e**) reference compound DosR-(Ursolic acid), fit on selected target protein were extracted from 200 ns MDS trajectories of different docked complexes.

The RMSD of protein Cα atoms across all complexes remained below 7.5 Å, indicating overall structural integrity. Among the ligands, M1, M3, and M4 exhibited final protein RMSD values of <4.8 Å, <6.4 Å, and <6.5 Å, respectively, demonstrating robust stability (Figure 4a,c,d). In contrast, M2 displayed a higher final protein RMSD (<9.5 Å), suggesting weaker binding (Figure 4b). The DosR-Ursolic acid complex exhibited an initial RMSD of 6 Å, peaking at <13.5 Å around 80 ns before stabilizing at <6.5 Å, implying delayed equilibration and potential instability requiring an extended simulation beyond 200 ns for conclusive assessment (Figure 4e).

**Figure 5:**
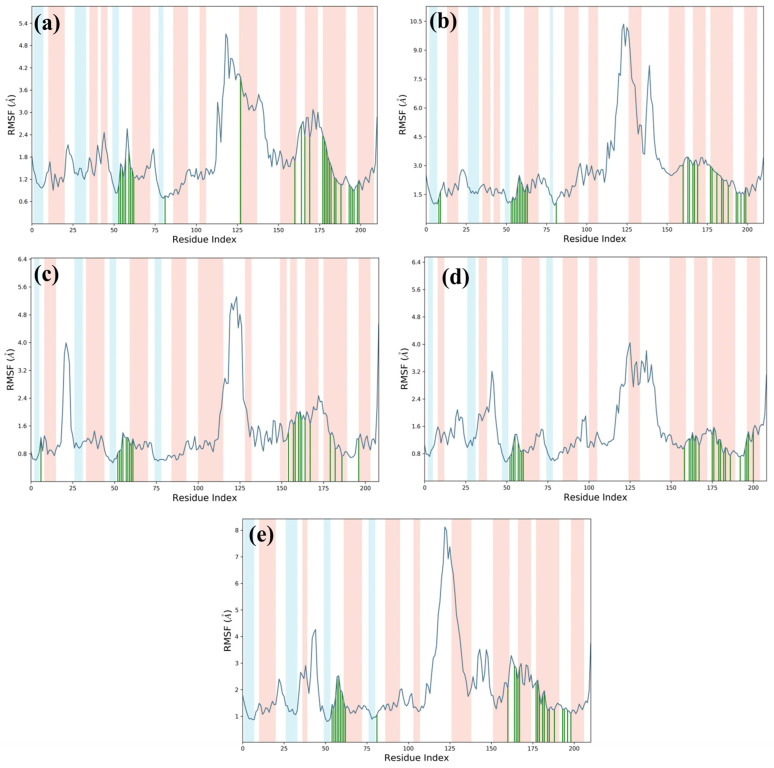
RMSF plot generated for the DosR protein docked with selected natural product compounds and reference molecule, i.e., (**a**) M1, (**b**) M2, (**c**) M3, (**d**) M4, and (**e**) reference compound (Ursolic acid).

**Figure 6:**
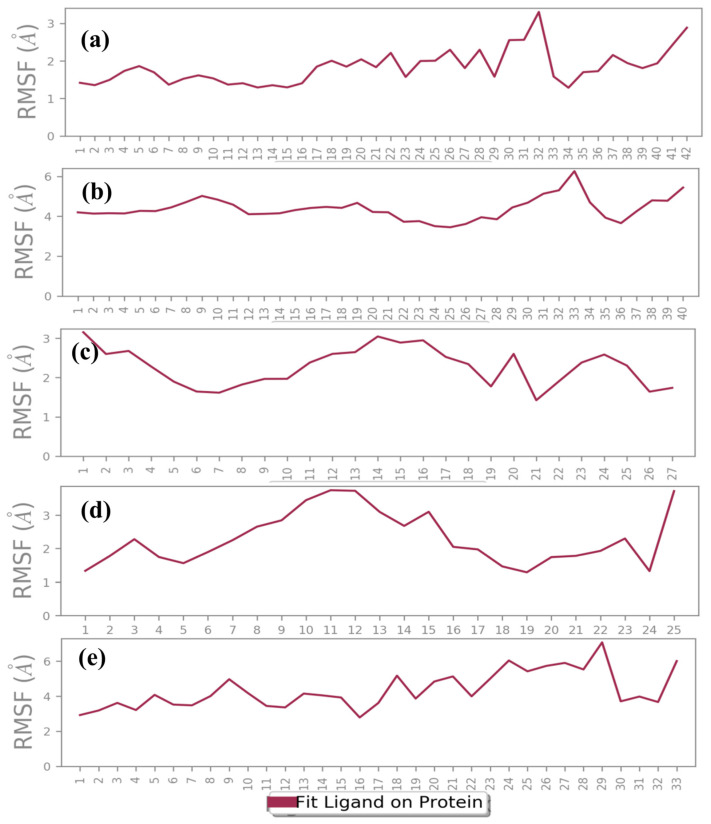
RMSF plot generated for the docked natural product compounds and reference molecule, i.e., (**a**) M1, (**b**) M2, (**c**) M3, (**d**) M4, and (**e**) reference compound (Ursolic acid), fit in the DosR protein during 200 ns MDS interval.

**Figure 7:**
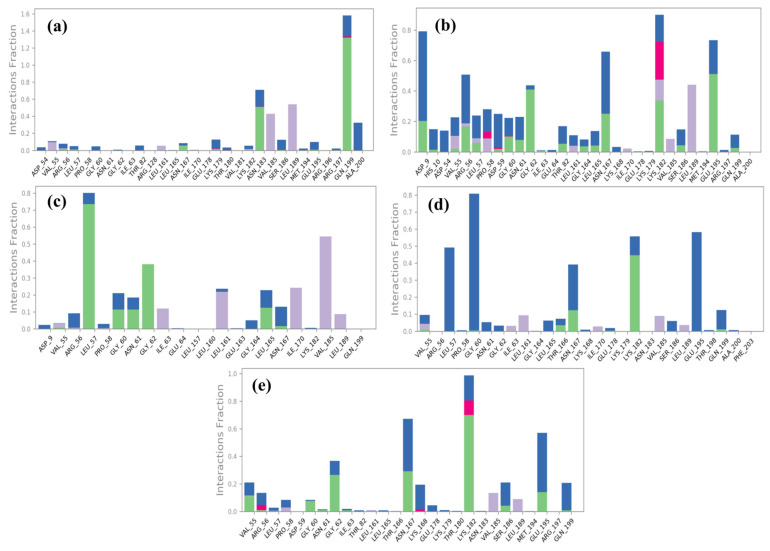
Protein–ligand interactions mapping (green: hydrogen bonding; grey: hydrophobic interaction; pink: ionic interaction; blue: water bridges) for DosR protein docked with selected natural product compounds, i.e., (**a**) DosR-M1, (**b**) DosR-M2, (**c**) DosR-M3, (**d**) DosR-M4, and (**e**) reference compound DosR (Ursolic acid), extracted from 200 ns Molecular dynamic simulations.

Ligand-specific RMSD analysis further differentiated stability trends. M1 maintained a stable trajectory (~3.2 Å) with a minor increase to 4.8 Å in the final 10 ns, indicative of slight conformational adjustments (Figure 4a). M3 exhibited minimal fluctuations (within 0.8 Å) after 30–40 ns, reflecting a highly stable binding pose (Figure 4c). M4 achieved equilibrium (~1 Å fluctuations) after 70 ns, whereas M2 demonstrated initial stability (3–3.5 Å) before destabilizing (~9 Å) beyond 150 ns (Figure 4b,d). Ursolic acid exhibited persistent instability, correlating with elevated protein RMSD, suggesting suboptimal binding (Figure 4e). The M3 complex stabilized within ~30 ns and maintained consistent RMSD, indicative of an energetically favorable binding mode. M4 required ~75 ns for convergence but remained stable thereafter. M1 exhibited a steady protein backbone but transient ligand deviations in the final 5–10 ns, suggesting dynamic binding. Conversely, M2 failed to stabilize, exhibiting pronounced fluctuations (Figure 4b), while Ursolic acid continued to display instability. These changes in Cα atoms were again supported by RMSF of the DosR protein in respective protein–ligand docked complexes, which exhibited equilibrium until the end of the 200 ns simulation interval but showed elevated RMSF in residues 110–150 across all complexes, suggesting localized structural flexibility and potentially influencing ligand-binding dynamics (Figure 5). RMSF analysis of ligands revealed that M1, M3, and M4 maintained atomic fluctuations below 4 Å, while M2 and Ursolic acid showed higher fluctuations (<6.5 Å) (Figure 6). Interaction profiling confirmed that M1, M3, and M4 formed extensive contacts with DosR, including hydrogen bonds with critical residues (e.g., Thr149, Ala150), hydrophobic interactions, and water bridges, surpassing or matching those of Ursolic acid. These interactions underpinned the enhanced stability of M1, M3, and M4 complexes (Figure 7).

In comparison to the reference compound Ursolic acid, selected compounds interacted with the range of residues of the DosR protein for the significant interactions during the 200 ns simulation. Selected compounds demonstrated more than one type of interaction viz. hydrogen bonding, hydrophobic interaction, ionic interaction, and water bridges formation with residues of the DosR protein (Figure 7). The DosR-M1 docked complex showed hydrogen bonding with (Gln199 and Asn183 residues), hydrophobic interaction (Val185 and Leu189), water bridges formation with residue Ala200 and with many residues for a shorter period of simulation time (<20% of total MDS time), and no ionic interaction was observed (Figure 7a). Likewise, the DosR-M2 complex exhibited hydrogen bond formation with (Asp9, Arg56, Asn167, Lys182, and Glu195 residues), hydrophobic interaction (Lys182 and Leu189 residues), ionic interaction (Pro58, Lys179), and water bridges formation with many residues (Figure 7b). Furthermore, the DosR- M3 docked complex depicted hydrogen bonding formation with (Leu57, Gly60, Asn61, Gly62, and Leu165), hydrophobic interaction (Val55, Ile63, Leu161, Ile170, Val185, and Leu189 residues), water bridges formation with many residues for shorter period of time, and no ionic interaction was observed (Figure 7c). Moreover, the DosR-M4 complex showed hydrogen bonding with (Asn167 and Lys182 residues), hydrophobic interaction with residues for a shorter period of time, and water bridges formation with (Leu57, Gly60, Asn167, and Glu195) (Figure 7d). On the other hand, reference complex DosR-Ursolic acid exhibited hydrogen bond formation with two residues only (Gly62, Asn167, and Lys182), and hydrophobic interaction with three residues through less than 10% of total MDS time. Meanwhile, Arg56 and Lys182 were noted for ionic interaction, and Asn167 and Glu195 were involved in water bridges formation (Figure 7e). Remarkably, many of these residues were also noted in the respective protein–ligand docked complexes (Table 2), which further support the stability of these screened compounds within the binding site of DosR protein.

**Figure 8:**
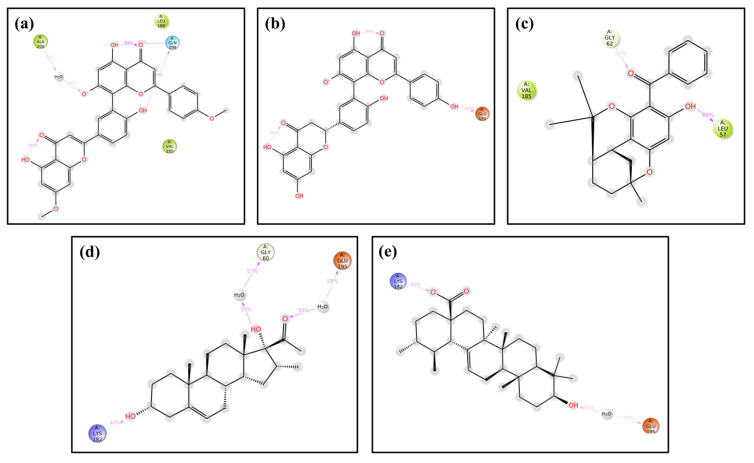
A detailed schematic representation of atomic interaction of ligand of natural product compounds and the reference molecule, i.e., (**a**) M1, (**b**) M2, (**c**) M3, (**d**) M4, and (**e**) reference compound (Ursolic acid), docked with DosR protein. On the chosen trajectory (0.00 to 200.01 nsec), interactions that occur more than 30.0% of the simulation period are displayed.

In ligand protein contacts diagrams, the binding site residues are consistently involved in ligand binding for more than 30% of MDS time in all docked complexes. This indicated the stability and specificity of interactions between the ligands and the DosR protein. The detailed percentages of interaction time with interacted residue are displayed in Figure 8 to provide further evidence for the binding behavior of each compound.

In the case of the DosR-M1 docked complex, the compound exhibited the formation of four H-bonds, with residues Ala200 (32%) and Gln199 (64% and 65%) of the simulation time (Figure 8a). In the DosR-M2 complex, three hydrogen bonds were noted, including a notable interaction with residue Lys182 for 51% of MDS time (Figure 8b). Furthermore, regarding the DosR-M3 complex, two H-bonds were observed, with residue Gly62 and Leu57 for 32% and 69% of total MDS time, respectively (Figure 8c). The DosR-M4 complex displayed two water-mediated hydrogen bonds with Gly60 and Glu195, persisting for 57% and 58% of the whole simulation interval, respectively. And one H-bonding was also observed with Lys182 for 44% of the MDS time (Figure 8d). Finally, the reference compound Ursolic acid showed two hydrogen bonds, a water-mediated hydrogen bond with Glu195 for 30% and a direct hydrogen bond with Lys182, observed for 46% of the total simulation time (Figure 8e).

These interactions emphasize their significant role in stability of complexes. The above analysis underscores the importance of these interactions, which were recorded for more than 30% of the whole simulation time, as depicted in the schematic representation of the ligand–protein contacts in Figure 8. Overall, the 200 ns MDS conclusively demonstrated that M3, M4, and M1 form stable complexes with DosR, outperforming M2 and Ursolic acid. M3’s rapid stabilization, minimal fluctuations, and robust interaction profile position it as the most promising therapeutic candidate. These findings, supported by extended simulations and detailed interaction analyses, highlight M3’s potential for further optimization in anti-tuberculosis drug development.

### 3.4. Binding Free Energy Analysis

**Figure 9:**
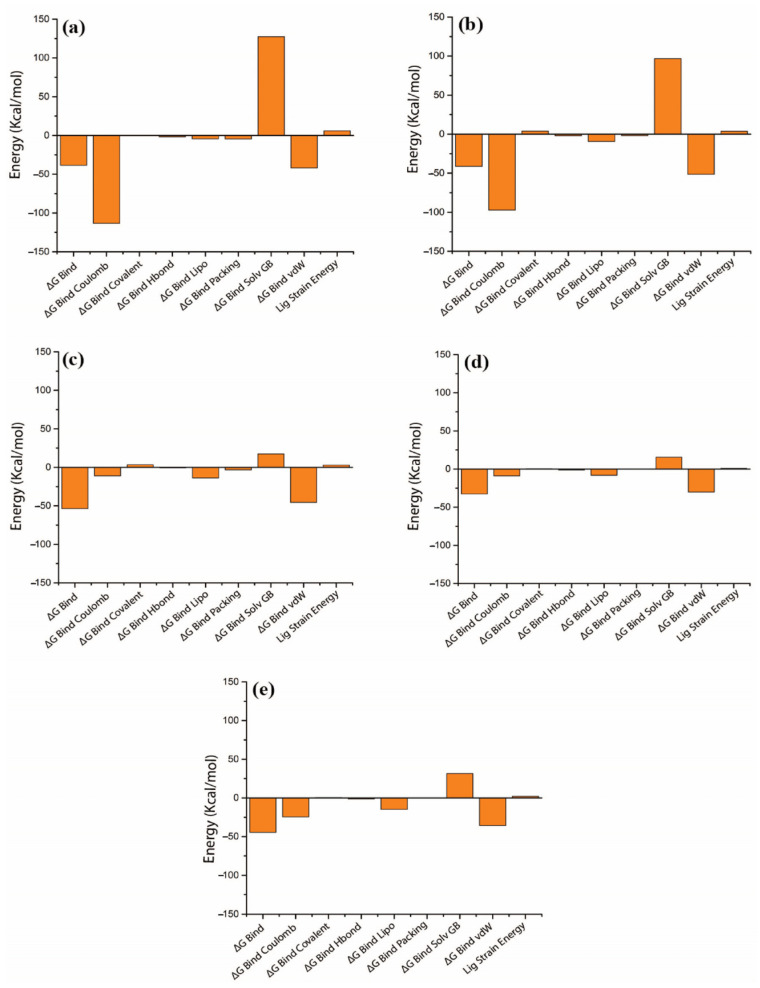
Calculated net binding free energy and energy components values for DosR protein complex with selected natural product compounds, i.e., (**a**) M1, (**b**) M2, (**c**) M3, (**d**) M4, and (**e**) reference compound (Ursolic acid), snapshots extracted from MDS trajectory.

The MM/GBSA result indicated that the binding free energy of M3 exhibited lowest value (ΔG Bind = −53.51 kcal/mol) than other compounds and the reference compound, indicating that the compound M3 shows stronger binding affinity than the reference compound and other selected compounds; this result is shown in Figure 9 and in Supplementary Table S2. The compound M2 showed binding free energy close to the reference compound ΔG Bind = −41.24 kcal/mol and ΔG Bind = −44.51 kcal/mol, respectively (Supplementary Table S2), while compound M1 indicated slightly higher and M4 exhibited highest binding free energy value, −38.47 kcal/mol and −32.59 kcal/mol, respectively, it indicated that compound M4 exhibits lowest binding affinity with DosR protein. ΔGBind Coulomb and ΔGBind vdW showed a significant contribution to the stability of each DosR protein–natural product compound complex. These findings suggest that compound M3 exhibited a significant affinity for the DosR protein (Figure 9 and in Supplementary Table S2). These values of binding free energy, therefore, supported the idea that natural-product-based compounds from the screening process could act as DosR inhibitors to prevent the latent TB infection.

## 4. Discussion

The primary focus of this study is to identify the potential natural-product-based inhibitors against the DosR protein of Mtb. Through SBVS of NP-lib database, four top ranked compounds were shortlisted and evaluated alongside reference compound i.e., Ursolic acid for further validation purpose. Among all, compound M3 was considered as the most potential compound, displaying the lowest binding free energy (ΔG Bind = −53.51 kcal/mol, with docking score −8.1 kcal/mol, and showed stable interactions over 200 ns MDS.

All these findings suggested that M3 has the capability to stably bind to DosR and interfere its function, which is essential for bacterial dormancy. It is noteworthy that the protein–ligand interaction profiling during MDS uncovered that M3 exhibited multiple H-bond, hydrophobic interactions, and water bridges formation, particularly with the residues Leu57, Gly60, Asn61, and Leu165. These interactions were formed for a substantial portion of simulation time. On comparing with Ursolic acid, M3 displayed superior performance. Ursolic acid exhibited low structural stability with high protein RMSD ~14 Å and weaker binding affinity with ΔGbind = −44.51 kcal/mol.

Similarly, in previous studies, Shanmuga Priya et al. [26] identified ZINC34198774 compound, which is considered a potential inhibitor of DosR regulon exhibiting docking score (−7.80 kcal/mol) and ΔGbind = −31.56 kcal/mol. The MDS analysis of this complex exhibited the RMSD to 0.55 nm (5.5 Å) at 5 ns, and then minor fluctuations were observed at 15 ns, 40 ns, and 75 ns, whereas the RMSD value of the complex remains the same throughout the simulation period [26]. Furthermore, Sharma et al. [27] identified a natural compound icariin as a potential inhibitor of the DosR protein, with a docking score of −5.92 kcal/mol and free binding energy ΔGbind = −52.96 kcal/mol. Furthermore, they compared their result with a previously identified inhibitor against DosR, HC104A, which had a docking score of −4.27 kcal/mol and free binding energy ΔGbind of −34.50 kcal/mol. The ligand HC104A and icariin depicted that low ligand RMSD lies between 1 and 4 Å. The DosR-icariin complex showed an increase in RMSD between 30 ns and 75 ns of the MDS with the rise of approx. 3–4 Å from its initial state, the RMSD again returned back to its original state after 75 ns of overall MDS. This is also supported by RMSF of the icariin-bound protein, which had an overall higher RMSF [27]. In comparison to these previously identified compounds, our best compound M3 exhibited the strongest binding with docking score greater than −8.1 kcal/mol, and ΔGbind −53.51 kcal/mol with DosR. The RMSD value (<6.8 Å) for the DosR-M3 docked complex was observed at the end of 200 ns. Analysis of both Protein RMSF and ligand RMSF showed that residues of protein and atoms of compound exhibited acceptable variations (<3 Å). These findings indicate that M3 may have stronger inhibitory potential than previously identified compounds, which makes it a potential lead for further investigation.

In spite of these findings, some limitations must be acknowledged. First, the whole study is totally based on computational methods. While these are very informative, they do not replicate the complexity of the biological system. Second, in vitro and in vivo validation was not performed. As such, to confirm its biological efficacy and cytotoxity profile, these validations have yet to be performed. Additionally, the main target of this study is DosR, whereas the dormancy of Mtb showed involvement of multiple targets and pathways. While DosR is a potential and validated target protein, a multi-target approach may be more effective in future therapeutic strategies.

Conclusively, M3 is a promising, natural-product-based compound that can bind potentially to the binding site of DosR protein and can be used for further validation studies by exploring it in in vitro and in vivo, which can give more transparent and clear results regarding the potentiality and efficacy of the identified compound. Moreover, this compound may lead to new therapeutics, which can be used to inhibit latent TB after evaluation through different experimental approaches in the future.

## 5. Conclusions

The current study emphasized the potential of natural-product-based compound M3 as an inhibitor against DosR protein, which is a key regulator in Mtb dormancy. Through computational approaches, including molecular docking, MDS, and MM/GBSA, compound M3 exhibited stronger binding affinity and stability compared to other compounds, including reference compound Ursolic acid. Furthermore, these results highlight the value of natural-product-based compounds in identifying lead compounds against LTBI. Findings from this computational study are encouraging, while further experimental validation, including in vitro and in vivo, is essential to validate the therapeutic potential of compound M3. Ultimately, targeting bacterial dormancy mechanisms may improve the existing TB treatment regimens and reduce disease persistence.

## Figures and Tables

**Table 1:** List of top four selected natural product compounds and reference compound docked against DosR protein of *Mycobacterium tuberculosis.*

Compound Name	Binding Energy (kcal/mol)	Redocking Score (kcal/mol)
M1	−8.5	−8.7
M2	−8.4	−8.5
M3	−8.2	−8.1
M4	−8.2	−8.2
reference compound (CID_64945 ursolic acid)	-	−6.2

**Table 2:** Molecular interaction profiling of top four selected docked complexes, and reference complex, i.e., (a) DosR-M1, (b) DosR-M2, (c) DosR-M3, (d) DosR-M4, and (e) reference complex (DosR-Ursolic acid).

Sr. No.	Drugs	Hydrogen-Bond	Hydrophobic	Polar	Negative	Positive	Glycine
a	DosR-M1	Arg197, Asn183	Val55, Leu161, Val185, Leu189, Met194	Asn61, Thr82, Asn167, Asn183, Ser186, Thr198, Gln199	Glu195	Arg56, Lys179, Lys182, Arg197	--
b	DosR-M2	-	Val55, Pro58, Leu161, Leu165, Val185, Leu189	His10, Asn61, Thr166, Asn167	Asp9	Arg56, Lys182	Gly60, Gly164
c	DosR-M3	Asn61	Val55, Leu57 Pro58, Leu161, Leu165, Ile170, Val185	Asn61, Thr166, Asn167	-	Arg56, Lys182	Gly60, Gly164
d	DosR-M4	Asn167	Val55, Leu57 Val185, Leu189	Asn61, Thr166, Asn167, Ser186	-	Arg56, Lys182	Gly60
e	DosR- Ursolic acid (CID_64945) (reference compound)	Asn61	Val55, Leu57, Pro58, Leu161, Leu165, Leu189, Val185,	Asn61, Thr166, Asn167, Ser186, Gln199	Asp59, Glu195	Arg56, Lys182	Gly164

## Data Availability

Data is contained within the article.

## Supplementary Materials

The following supporting information can be downloaded at: https://www.mdpi.com/article/10.3390/arm93030019/s1. Table S1: List of natural product compounds with their binding energy extracted from structure-based virtual screening result using MTiOpenScreen web server; Table S2: Calculated net Binding free energy and energy components values for DosR protein complexes with natural compounds snapshots collected from MD simulation trajectories.
