# Supplementary material for: Harnessing Natural Product Compounds to Target Dormancy Survival Regulator (DosR) in Latent Tuberculosis Infection (LTBI): An In Silico Strategy Against Dormancy
Source: Adv Respir Med. 2025 Jun 16;93(3):19. doi: 10.3390/arm93030019 (PMC12190169; doi:10.3390/arm93030019)
Supplement: Supplementary file 1 [file arm-93-00019-s001.zip › arm-3585714-supplementary.pdf]

Supplementary file

Table S1: List of natural product compounds with their binding energy extracted from structure-based virtual screening result using MTiOpenScreen web server.

Compound	Model ID	Energy (kcal/mol)
ZINC000003594862	1	-8.5
ZINC000059779788	1	-8.4
ZINC000230017540	1	-8.2
ZINC000253501597	1	-8.2
ZINC000038140884	1	-8.1
ZINC000002573069	1	-8.1
ZINC000005356864	1	-8
ZINC000015205931	1	-8
ZINC000015205928	1	-8
ZINC000004654620	1	-7.9
ZINC000014766825	1	-7.9
ZINC000253497719	1	-7.9
ZINC000001690209	1	-7.9
ZINC000000086467	1	-7.9
ZINC000038140891	1	-7.8
ZINC000253503169	1	-7.8

ZINC000014887151	1	-7.8
ZINC000038140889	1	-7.8
ZINC000098052524	1	-7.7
ZINC000253388536	1	-7.7
ZINC000009210416	1	-7.7
ZINC000031158814	1	-7.7
ZINC000014887157	1	-7.7
ZINC000044404209	1	-7.7
ZINC000013485410	1	-7.7
ZINC000038563736	1	-7.6
ZINC000005849312	1	-7.6
ZINC000001872259	1	-7.6
ZINC000013383651	1	-7.6
ZINC000003947429	1	-7.6
ZINC000252517497	1	-7.6
ZINC000085602366	1	-7.6
ZINC000524731672	1	-7.6
ZINC000004349417	1	-7.6
ZINC000014887147	1	-7.6
ZINC000013424697	1	-7.6
ZINC000253497718	1	-7.5

ZINC000072169036	1	-7.5
ZINC000006528354	1	-7.5
ZINC000001562070	1	-7.5
ZINC000003861134	1	-7.5
ZINC000014824336	1	-7.5
ZINC000253503170	1	-7.5
ZINC000000001419	1	-7.5
ZINC000031158807	1	-7.5
ZINC000049841244	1	-7.5
ZINC000004023302	1	-7.5
ZINC000004096258	1	-7.5
ZINC000004731298	1	-7.5
ZINC000299817665	1	-7.5
ZINC000040394781	1	-7.5
ZINC000253504517	1	-7.5
ZINC000008143604	1	-7.5
ZINC000000828203	1	-7.4
ZINC000014824338	1	-7.4
ZINC000253388708	1	-7.4
ZINC000014727561	1	-7.4
ZINC000248012330	1	-7.4

ZINC000038143822	1	-7.4
ZINC000002033819	1	-7.4
ZINC000000603253	1	-7.4
ZINC000005158963	1	-7.4
ZINC000034265086	1	-7.4
ZINC000013783428	1	-7.4
ZINC000253497332	1	-7.4
ZINC000022061725	1	-7.4
ZINC000245205019	1	-7.4
ZINC000118913600	1	-7.4
ZINC000002121515	1	-7.4
ZINC000004023301	1	-7.4
ZINC000137376946	1	-7.4
ZINC000028537536	1	-7.4
ZINC000390822360	1	-7.4
ZINC000004252704	1	-7.3
ZINC000005195795	1	-7.3
ZINC000067910611	1	-7.3
ZINC000013816219	1	-7.3
ZINC000096023661	1	-7.3
ZINC000000031991	1	-7.3

ZINC000003873159	1	-7.3
ZINC000049181130	1	-7.3
ZINC000002554900	1	-7.3
ZINC000248012342	1	-7.3
ZINC000000517415	1	-7.3
ZINC000003176041	1	-7.3
ZINC000253530470	1	-7.3
ZINC000253617029	1	-7.3
ZINC000002033818	1	-7.3
ZINC000014637754	1	-7.3
ZINC000038140890	1	-7.3
ZINC000014814927	1	-7.3
ZINC000005854524	1	-7.3
ZINC000003953891	1	-7.3
ZINC000004102435	1	-7.3
ZINC000059729955	1	-7.3
ZINC000253388709	1	-7.3
ZINC000014819752	1	-7.3
ZINC000014758732	1	-7.3
ZINC000253497664	1	-7.3
ZINC000014824332	1	-7.2

ZINC000003861133	1	-7.2
ZINC000095914837	1	-7.2
ZINC000253499207	1	-7.2
ZINC000014819753	1	-7.2
ZINC000253531992	1	-7.2
ZINC000012890900	1	-7.2
ZINC000001022034	1	-7.2
ZINC000169717638	1	-7.2
ZINC000003872147	1	-7.2
ZINC000003875408	1	-7.2
ZINC000014688352	1	-7.2
ZINC000253499148	1	-7.2
ZINC000252441490	1	-7.2
ZINC000229938097	1	-7.2
ZINC000005512974	1	-7.2
ZINC000253497717	1	-7.2
ZINC000253503172	1	-7.2
ZINC000006018563	1	-7.2
ZINC000006092939	1	-7.2
ZINC000253530471	1	-7.2
ZINC000252517498	1	-7.2

ZINC000257360793	1	-7.2
ZINC000033831794	1	-7.2
ZINC000253499146	1	-7.2
ZINC000004349478	1	-7.1
ZINC000002083344	1	-7.1
ZINC000096111561	1	-7.1
ZINC000253387985	1	-7.1
ZINC000253388707	1	-7.1
ZINC000038140885	1	-7.1
ZINC000001637085	1	-7.1
ZINC000252483567	1	-7.1
ZINC000253530469	1	-7.1
ZINC000238749956	1	-7.1
ZINC000096111560	1	-7.1
ZINC000253388706	1	-7.1
ZINC000000119434	1	-7.1
ZINC000253387984	1	-7.1
ZINC000299817890	1	-7.1
ZINC000002570158	1	-7.1
ZINC000005640448	1	-7.1
ZINC000004000041	1	-7.1

ZINC000004098804	1	-7.1
ZINC000253497666	1	-7.1
ZINC000253499149	1	-7.1
ZINC000087493014	1	-7.1
ZINC000257360792	1	-7.1
ZINC000006017925	1	-7.1
ZINC000075906045	1	-7.1
ZINC000014820619	1	-7.1
ZINC000248008265	1	-7.1
ZINC000000900115	1	-7.1
ZINC000252441407	1	-7.1
ZINC000053195720	1	-7.1
ZINC000049181121	1	-7.1
ZINC000031160738	1	-7.1
ZINC000253390010	1	-7.1
ZINC000031156322	1	-7.1
ZINC000011865165	1	-7.1
ZINC000031165745	1	-7.1
ZINC000004906153	1	-7.1
ZINC000026472270	1	-7
ZINC000014711629	1	-7

ZINC000253530472	1	-7
ZINC000003947427	1	-7
ZINC000006536273	1	-7
ZINC000254302318	1	-7
ZINC000005158921	1	-7
ZINC000070691661	1	-7
ZINC000004023126	1	-7
ZINC000037538575	1	-7
ZINC000005998555	1	-7
ZINC000015121008	1	-7
ZINC000254302320	1	-7
ZINC000000518473	1	-7
ZINC000005732375	1	-7
ZINC000299817465	1	-7
ZINC000070691536	1	-7
ZINC000006092955	1	-7
ZINC000059728627	1	-7
ZINC000253498823	1	-7
ZINC000059402085	1	-7
ZINC000252516283	1	-7
ZINC000390822361	1	-7

ZINC000000338284	1	-7
ZINC000004082225	1	-7
ZINC000253531991	1	-7
ZINC000070691538	1	-7
ZINC000044582844	1	-7
ZINC000000899938	1	-7
ZINC000003815418	1	-7
ZINC000043620416	1	-7
ZINC000096023667	1	-7
ZINC000070691533	1	-7
ZINC000253388412	1	-7
ZINC000001711825	1	-7
ZINC000013507959	1	-7
ZINC000005447704	1	-7
ZINC000004175638	1	-7
ZINC000014437288	1	-7
ZINC000014952116	1	-6.9
ZINC000253497333	1	-6.9
ZINC000014728481	1	-6.9
ZINC000100306588	1	-6.9
ZINC000004037920	1	-6.9

ZINC000247855168	1	-6.9
ZINC000253497665	1	-6.9
ZINC000095933385	1	-6.9
ZINC000253497716	1	-6.9
ZINC000008918451	1	-6.9
ZINC000064859315	1	-6.9
ZINC000005838160	1	-6.9
ZINC000013383630	1	-6.9
ZINC000253497993	1	-6.9
ZINC000014887154	1	-6.9
ZINC000013340662	1	-6.9
ZINC000015117976	1	-6.9
ZINC000253499604	1	-6.9
ZINC000253497713	1	-6.9
ZINC000014824334	1	-6.9
ZINC000036057337	1	-6.9
ZINC000014410651	1	-6.9
ZINC000005732527	1	-6.9
ZINC000044362063	1	-6.9
ZINC000070691648	1	-6.9
ZINC000142857948	1	-6.9

ZINC000033505346	1	-6.9
ZINC000003157052	1	-6.9
ZINC000012890906	1	-6.9
ZINC000013374325	1	-6.9
ZINC000000338331	1	-6.9
ZINC000015121009	1	-6.9
ZINC000252516281	1	-6.9
ZINC000255231862	1	-6.9
ZINC000001433941	1	-6.9
ZINC000003947430	1	-6.9
ZINC000011615757	1	-6.9
ZINC000004027344	1	-6.9
ZINC000003881360	1	-6.9
ZINC000004369665	1	-6.9
ZINC000014441557	1	-6.9
ZINC000026977303	1	-6.9
ZINC000013374323	1	-6.8
ZINC000005854532	1	-6.8
ZINC000075270370	1	-6.8
ZINC000014760237	1	-6.8
ZINC000005273905	1	-6.8

ZINC000005732241	1	-6.8
ZINC000005665087	1	-6.8
ZINC000005732268	1	-6.8
ZINC000059770263	1	-6.8
ZINC000004731322	1	-6.8
ZINC000002121517	1	-6.8
ZINC000003869900	1	-6.8
ZINC000253390009	1	-6.8
ZINC000106611619	1	-6.8
ZINC000008214766	1	-6.8
ZINC000001615142	1	-6.8
ZINC000004097876	1	-6.8
ZINC000253497610	1	-6.8
ZINC000014728438	1	-6.8
ZINC000005132927	1	-6.8
ZINC000095099608	1	-6.8
ZINC000014642994	1	-6.8
ZINC000100037384	1	-6.8
ZINC000004731321	1	-6.8
ZINC000030730478	1	-6.8
ZINC000031459237	1	-6.8

ZINC000067913388	1	-6.8
ZINC000248089500	1	-6.8
ZINC000299817840	1	-6.8
ZINC000238809247	1	-6.8
ZINC000008730438	1	-6.8
ZINC000253530362	1	-6.8
ZINC000252505842	1	-6.8
ZINC000015274349	1	-6.8
ZINC000003870887	1	-6.8
ZINC000031170081	1	-6.8
ZINC000070691657	1	-6.7
ZINC000016889922	1	-6.7
ZINC000004081132	1	-6.7
ZINC000013607514	1	-6.7
ZINC000070454025	1	-6.7
ZINC000013302315	1	-6.7
ZINC000253497711	1	-6.7
ZINC000004098747	1	-6.7
ZINC000014758734	1	-6.7
ZINC000252477564	1	-6.7
ZINC000012362075	1	-6.7

ZINC000004404409	1	-6.7
ZINC000253497998	1	-6.7
ZINC000001645468	1	-6.7
ZINC000015258073	1	-6.7
ZINC000105153378	1	-6.7
ZINC000038140888	1	-6.7
ZINC000100060215	1	-6.7
ZINC000011818030	1	-6.7
ZINC000036375281	1	-6.7
ZINC000001000254	1	-6.7
ZINC000005761187	1	-6.7
ZINC000000278217	1	-6.7
ZINC000038139805	1	-6.7
ZINC000013383333	1	-6.7
ZINC000087492983	1	-6.7
ZINC000253497334	1	-6.7
ZINC000013339945	1	-6.7
ZINC000096095464	1	-6.7
ZINC000001643072	1	-6.7
ZINC000004349592	1	-6.7
ZINC000253497714	1	-6.7

ZINC000049823049	1	-6.7
ZINC000253388414	1	-6.7
ZINC000253387986	1	-6.7
ZINC000014410646	1	-6.7
ZINC000255209314	1	-6.7
ZINC000031155321	1	-6.7
ZINC000014557640	1	-6.7
ZINC000253529982	1	-6.7
ZINC000000899166	1	-6.7
ZINC000038563738	1	-6.7
ZINC000004098306	1	-6.7
ZINC000247855174	1	-6.7
ZINC000077031606	1	-6.7
ZINC000000338283	1	-6.7
ZINC000257603519	1	-6.7
ZINC000002983276	1	-6.7
ZINC000012888734	1	-6.7
ZINC000004027521	1	-6.6
ZINC000013838742	1	-6.6
ZINC000005158566	1	-6.6
ZINC000003977995	1	-6.6

ZINC000008551637	1	-6.6
ZINC000013485421	1	-6.6
ZINC000085576447	1	-6.6
ZINC000000538127	1	-6.6
ZINC000013302310	1	-6.6
ZINC000003869898	1	-6.6
ZINC000011615762	1	-6.6
ZINC000000035528	1	-6.6
ZINC000252516942	1	-6.6
ZINC000253390006	1	-6.6
ZINC000013485423	1	-6.6
ZINC000075270374	1	-6.6
ZINC000004098607	1	-6.6
ZINC000003649911	1	-6.6
ZINC000005158973	1	-6.6
ZINC000252516282	1	-6.6
ZINC000067912742	1	-6.6
ZINC000138560360	1	-6.6
ZINC000012888727	1	-6.6
ZINC000238809538	1	-6.6
ZINC000000035529	1	-6.6

ZINC000006018635	1	-6.6
ZINC000003643476	1	-6.6
ZINC000028539634	1	-6.6
ZINC000013374324	1	-6.6
ZINC000033832448	1	-6.6
ZINC000004064009	1	-6.6
ZINC000015208646	1	-6.6
ZINC000014410655	1	-6.6
ZINC000031169794	1	-6.6
ZINC000015274352	1	-6.6
ZINC000008829452	1	-6.6
ZINC000004097489	1	-6.6
ZINC000004117655	1	-6.6
ZINC000004082214	1	-6.5
ZINC000252286706	1	-6.5
ZINC000012153561	1	-6.5
ZINC000013374008	1	-6.5
ZINC000070455246	1	-6.5
ZINC000097971614	1	-6.5
ZINC000034612334	1	-6.5
ZINC000253502228	1	-6.5

ZINC000014489321	1	-6.5
ZINC000253389930	1	-6.5
ZINC000096023629	1	-6.5
ZINC000039014143	1	-6.5
ZINC000067903629	1	-6.5
ZINC000252441488	1	-6.5
ZINC000014642730	1	-6.5
ZINC000253497995	1	-6.5
ZINC000001726300	1	-6.5
ZINC000106007458	1	-6.5
ZINC000031155312	1	-6.5
ZINC000253502229	1	-6.5
ZINC000254302317	1	-6.5
ZINC000253498755	1	-6.5
ZINC000001095283	1	-6.5
ZINC000004612836	1	-6.5
ZINC000013485420	1	-6.5
ZINC000003613216	1	-6.5
ZINC000253390008	1	-6.5
ZINC000005648525	1	-6.5
ZINC000004026203	1	-6.5

ZINC000002585423	1	-6.5
ZINC000001016260	1	-6.5
ZINC000005761186	1	-6.5
ZINC000253530726	1	-6.5
ZINC000253502230	1	-6.5
ZINC000253499203	1	-6.5
ZINC000012868385	1	-6.5
ZINC000238809330	1	-6.5
ZINC000013429289	1	-6.5
ZINC000149170493	1	-6.5
ZINC000015208649	1	-6.5
ZINC000003881961	1	-6.5
ZINC000255269628	1	-6.5
ZINC000248070698	1	-6.5
ZINC000000481564	1	-6.5
ZINC000000000052	1	-6.5
ZINC000252517496	1	-6.5
ZINC000004027641	1	-6.5
ZINC000004478069	1	-6.5
ZINC000252441491	1	-6.5
ZINC000100009280	1	-6.5

ZINC000000051924	1	-6.5
ZINC000067912695	1	-6.5
ZINC000012888720	1	-6.5
ZINC000253500806	1	-6.5
ZINC000252477565	1	-6.5
ZINC000001588843	1	-6.5
ZINC000049181127	1	-6.5
ZINC000004081360	1	-6.5
ZINC000252441409	1	-6.5
ZINC000013411981	1	-6.4
ZINC000013307187	1	-6.4
ZINC000013548872	1	-6.4
ZINC000019796061	1	-6.4
ZINC000031155655	1	-6.4
ZINC000000477938	1	-6.4
ZINC000070691659	1	-6.4
ZINC000003833872	1	-6.4
ZINC000253390544	1	-6.4
ZINC000003947428	1	-6.4
ZINC000034546884	1	-6.4
ZINC000043763825	1	-6.4

ZINC000006036807	1	-6.4
ZINC000138700111	1	-6.4
ZINC000004000040	1	-6.4
ZINC000015274351	1	-6.4
ZINC000238731755	1	-6.4
ZINC000005567798	1	-6.4
ZINC000238808778	1	-6.4
ZINC000038139804	1	-6.4
ZINC000006745365	1	-6.4
ZINC000002983277	1	-6.4
ZINC000248070690	1	-6.4
ZINC000031164167	1	-6.4
ZINC000000052858	1	-6.4
ZINC000245240534	1	-6.4
ZINC000253497994	1	-6.4
ZINC000253531993	1	-6.4
ZINC000013509501	1	-6.4
ZINC000004273373	1	-6.4
ZINC000000899848	1	-6.4
ZINC000006793326	1	-6.4
ZINC000013429291	1	-6.4

ZINC000138700005	1	-6.4
ZINC000253504510	1	-6.4
ZINC000049181124	1	-6.4
ZINC000038143820	1	-6.4
ZINC000028968101	1	-6.4
ZINC000026832394	1	-6.4
ZINC000014437227	1	-6.4
ZINC000253497992	1	-6.4
ZINC000004046820	1	-6.4
ZINC000159906818	1	-6.4
ZINC000004000056	1	-6.4
ZINC000006793327	1	-6.4
ZINC000252477566	1	-6.4
ZINC000031156316	1	-6.4
ZINC000012875616	1	-6.4
ZINC000020805004	1	-6.4
ZINC000169721061	1	-6.4
ZINC000013429285	1	-6.4
ZINC000067912718	1	-6.3
ZINC000031156142	1	-6.3
ZINC000253388535	1	-6.3

ZINC000015165008	1	-6.3
ZINC000096023772	1	-6.3
ZINC000004517149	1	-6.3
ZINC000252516941	1	-6.3
ZINC000070691794	1	-6.3
ZINC000058576036	1	-6.3
ZINC000034114040	1	-6.3
ZINC000067913661	1	-6.3
ZINC000004027386	1	-6.3
ZINC000253500939	1	-6.3
ZINC000003871987	1	-6.3
ZINC000299817767	1	-6.3
ZINC000004262223	1	-6.3
ZINC000000439867	1	-6.3
ZINC000095785874	1	-6.3
ZINC000013485422	1	-6.3
ZINC000004097850	1	-6.3
ZINC000253389929	1	-6.3
ZINC000000035526	1	-6.3
ZINC000100059326	1	-6.3
ZINC000004081136	1	-6.3

ZINC000005998211	1	-6.3
ZINC000001722927	1	-6.3
ZINC000036368151	1	-6.3
ZINC000252441406	1	-6.3
ZINC000031155650	1	-6.3
ZINC000004097817	1	-6.3
ZINC000013340319	1	-6.3
ZINC000253506262	1	-6.3
ZINC000033831904	1	-6.3
ZINC000096023790	1	-6.3
ZINC000067902540	1	-6.3
ZINC000000481563	1	-6.3
ZINC000006070275	1	-6.3
ZINC000248070694	1	-6.3
ZINC000002106549	1	-6.3
ZINC000247999548	1	-6.3
ZINC000004097893	1	-6.3
ZINC000005761412	1	-6.3
ZINC000035049228	1	-6.3
ZINC000253530364	1	-6.3
ZINC000067902988	1	-6.3

ZINC000253390081	1	-6.3
ZINC000020593688	1	-6.3
ZINC000004098262	1	-6.3
ZINC000067902828	1	-6.3
ZINC000105426804	1	-6.3
ZINC000261493072	1	-6.3
ZINC000067913644	1	-6.3
ZINC000034319582	1	-6.3
ZINC000039010260	1	-6.3
ZINC000253389027	1	-6.3
ZINC000253504507	1	-6.3
ZINC000006040344	1	-6.3
ZINC000146803900	1	-6.2
ZINC000101669329	1	-6.2
ZINC000005854594	1	-6.2
ZINC000085539971	1	-6.2
ZINC000004097188	1	-6.2
ZINC000159906407	1	-6.2
ZINC000159906551	1	-6.2
ZINC000013838615	1	-6.2
ZINC000000136036	1	-6.2

ZINC000014651078	1	-6.2
ZINC000015057917	1	-6.2
ZINC000013305564	1	-6.2
ZINC000136914527	1	-6.2
ZINC000253389928	1	-6.2
ZINC000252505843	1	-6.2
ZINC000248008261	1	-6.2
ZINC000004097583	1	-6.2
ZINC000035456370	1	-6.2
ZINC000253389931	1	-6.2
ZINC000013111092	1	-6.2
ZINC000034319583	1	-6.2
ZINC000096023863	1	-6.2
ZINC000253498824	1	-6.2
ZINC000253500840	1	-6.2
ZINC000011843017	1	-6.2
ZINC000253500936	1	-6.2
ZINC000000895263	1	-6.2
ZINC000238774071	1	-6.2
ZINC000150447412	1	-6.2
ZINC000013377893	1	-6.2

ZINC000031164163	1	-6.2
ZINC000004027640	1	-6.2
ZINC000004478068	1	-6.2
ZINC000000339740	1	-6.2
ZINC000003881960	1	-6.2
ZINC000044351843	1	-6.2
ZINC000253529984	1	-6.2
ZINC000253497609	1	-6.2
ZINC000031164159	1	-6.2
ZINC000087492966	1	-6.2
ZINC000015121972	1	-6.2
ZINC000032024272	1	-6.2
ZINC000253389264	1	-6.2
ZINC000049889356	1	-6.2
ZINC000253500929	1	-6.2
ZINC000005761413	1	-6.2
ZINC000004478070	1	-6.2
ZINC000026826077	1	-6.2
ZINC000027103414	1	-6.2
ZINC000004228284	1	-6.2
ZINC000253390543	1	-6.1

ZINC000038358415	1	-6.1
ZINC000253530363	1	-6.1
ZINC000034955328	1	-6.1
ZINC000000897924	1	-6.1
ZINC000253500807	1	-6.1
ZINC000000434672	1	-6.1
ZINC000008829484	1	-6.1
ZINC000013480529	1	-6.1
ZINC000096023811	1	-6.1
ZINC000031155660	1	-6.1
ZINC000031155424	1	-6.1
ZINC000004090458	1	-6.1
ZINC000247999543	1	-6.1
ZINC000031156152	1	-6.1
ZINC000031459294	1	-6.1
ZINC000252516940	1	-6.1
ZINC000034430431	1	-6.1
ZINC000067903362	1	-6.1
ZINC000253497800	1	-6.1
ZINC000003776540	1	-6.1
ZINC000096023837	1	-6.1

ZINC000004097713	1	-6.1
ZINC000254302319	1	-6.1
ZINC000100037376	1	-6.1
ZINC000013377891	1	-6.1
ZINC000253534341	1	-6.1
ZINC000013545957	1	-6.1
ZINC000225459406	1	-6.1
ZINC000252441489	1	-6.1
ZINC000034169935	1	-6.1
ZINC000253497612	1	-6.1
ZINC000001542895	1	-6.1
ZINC000013305561	1	-6.1
ZINC000253506247	1	-6.1
ZINC000031155594	1	-6.1
ZINC000001646488	1	-6.1
ZINC000040576475	1	-6.1
ZINC000004026432	1	-6.1
ZINC000034513670	1	-6.1
ZINC000238809655	1	-6.1
ZINC000031155418	1	-6.1
ZINC000253390541	1	-6.1

ZINC000014980286	1	-6.1
ZINC000004273370	1	-6.1
ZINC000031350059	1	-6.1
ZINC000013374322	1	-6.1
ZINC000097971734	1	-6
ZINC000005765855	1	-6
ZINC000253497997	1	-6
ZINC000095909891	1	-6
ZINC000253500931	1	-6
ZINC000000140178	1	-6
ZINC000031164155	1	-6
ZINC000004087038	1	-6
ZINC000027550897	1	-6
ZINC000002444172	1	-6
ZINC000006067010	1	-6
ZINC000253530361	1	-6
ZINC000253389263	1	-6
ZINC000038143766	1	-6
ZINC000253390080	1	-6
ZINC000049781954	1	-6
ZINC000061948698	1	-6

ZINC000000338330	1	-6
ZINC000013507846	1	-6
ZINC000000689725	1	-6
ZINC000005854293	1	-6
ZINC000098214437	1	-6
ZINC000253500838	1	-6
ZINC000253500933	1	-6
ZINC000017111150	1	-6
ZINC000059589003	1	-6
ZINC000252515034	1	-6
ZINC000101025375	1	-6
ZINC000001872131	1	-6
ZINC000067910656	1	-6
ZINC000253500839	1	-6
ZINC000253497712	1	-6
ZINC000067903504	1	-6
ZINC000008789489	1	-6
ZINC000253506252	1	-6
ZINC000013541615	1	-6
ZINC000013370395	1	-6
ZINC000013550925	1	-6

ZINC000253497769	1	-6
ZINC000070691553	1	-6
ZINC000034012896	1	-6
ZINC000008220175	1	-6
ZINC000253388413	1	-6
ZINC000031170087	1	-6
ZINC000001734352	1	-6
ZINC000035271669	1	-6
ZINC000001646489	1	-6
ZINC000096023899	1	-6
ZINC000003978827	1	-6
ZINC000253534343	1	-6
ZINC000253388415	1	-6
ZINC000001872258	1	-6
ZINC000100037386	1	-6
ZINC000031156147	1	-6
ZINC000059064833	1	-6
ZINC000004023124	1	-6
ZINC000001595957	1	-5.9
ZINC000225459438	1	-5.9
ZINC000004478067	1	-5.9

ZINC000253497999	1	-5.9
ZINC000006522158	1	-5.9
ZINC000252441408	1	-5.9
ZINC000040385796	1	-5.9
ZINC000248007364	1	-5.9
ZINC000095910344	1	-5.9
ZINC000253534708	1	-5.9
ZINC000004716552	1	-5.9
ZINC000059065484	1	-5.9
ZINC000248341256	1	-5.9
ZINC000013480530	1	-5.9
ZINC000253498754	1	-5.9
ZINC000006069011	1	-5.9
ZINC000253498822	1	-5.9
ZINC000039390775	1	-5.9
ZINC000049601451	1	-5.9
ZINC000000336322	1	-5.9
ZINC000032296643	1	-5.9
ZINC000245224268	1	-5.9
ZINC000049538671	1	-5.9
ZINC000004517154	1	-5.9

ZINC000229903602	1	-5.9
ZINC000008762126	1	-5.9
ZINC000031158109	1	-5.9
ZINC000083260785	1	-5.9
ZINC000004654776	1	-5.9
ZINC000004087035	1	-5.9
ZINC000159906680	1	-5.9
ZINC000004081361	1	-5.9
ZINC000004273372	1	-5.9
ZINC000027550862	1	-5.9
ZINC000003881999	1	-5.9
ZINC000253500804	1	-5.9
ZINC000085539966	1	-5.8
ZINC000072320389	1	-5.8
ZINC000031158115	1	-5.8
ZINC000098214409	1	-5.8
ZINC000002507487	1	-5.8
ZINC000034955330	1	-5.8
ZINC000000525698	1	-5.8
ZINC000100963287	1	-5.8
ZINC000252515032	1	-5.8

ZINC000031155646	1	-5.8
ZINC000095639161	1	-5.8
ZINC000002144927	1	-5.8
ZINC000239073254	1	-5.8
ZINC000239073257	1	-5.8
ZINC000096023666	1	-5.8
ZINC000036370614	1	-5.8
ZINC000031164787	1	-5.8
ZINC000085555528	1	-5.8
ZINC000100043983	1	-5.8
ZINC000253529983	1	-5.8
ZINC000241132997	1	-5.8
ZINC000004273371	1	-5.8
ZINC000136358084	1	-5.8
ZINC000031164525	1	-5.8
ZINC000013460752	1	-5.8
ZINC000253390546	1	-5.8
ZINC000035410506	1	-5.8
ZINC000015147908	1	-5.8
ZINC000013377892	1	-5.8
ZINC000031164783	1	-5.8

ZINC000100779034	1	-5.8
ZINC000252515033	1	-5.8
ZINC000150664131	1	-5.8
ZINC000031164794	1	-5.8
ZINC000002558134	1	-5.8
ZINC000299817804	1	-5.8
ZINC000008951991	1	-5.8
ZINC000067912745	1	-5.8
ZINC000299817832	1	-5.8
ZINC000253388537	1	-5.8
ZINC000004064007	1	-5.8
ZINC000067913655	1	-5.8
ZINC000248089453	1	-5.7
ZINC000004081362	1	-5.7
ZINC000035410508	1	-5.7
ZINC000031164791	1	-5.7
ZINC000253534711	1	-5.7
ZINC000014517491	1	-5.7
ZINC000013311350	1	-5.7
ZINC000103890561	1	-5.7
ZINC000002024082	1	-5.7

ZINC000118912966	1	-5.7
ZINC000000189939	1	-5.7
ZINC000097971733	1	-5.7
ZINC000095410473	1	-5.7
ZINC000003789195	1	-5.7
ZINC000013311346	1	-5.7
ZINC000049538670	1	-5.7
ZINC000000392595	1	-5.7
ZINC000107434573	1	-5.7
ZINC000004096264	1	-5.7
ZINC000005024269	1	-5.7
ZINC000031165817	1	-5.7
ZINC000005369099	1	-5.7
ZINC000035410507	1	-5.7
ZINC000242437516	1	-5.7
ZINC000248105733	1	-5.7
ZINC000004097122	1	-5.7
ZINC000253389367	1	-5.7
ZINC000006495303	1	-5.7
ZINC000022066763	1	-5.7
ZINC000015270770	1	-5.7

ZINC000252515031	1	-5.7
ZINC000225257462	1	-5.7
ZINC000003882000	1	-5.7
ZINC000004081365	1	-5.7
ZINC000035410505	1	-5.7
ZINC000001037159	1	-5.7
ZINC000004026431	1	-5.7
ZINC000000386238	1	-5.6
ZINC000015270842	1	-5.6
ZINC000005722186	1	-5.6
ZINC000012879245	1	-5.6
ZINC000019517337	1	-5.6
ZINC000000062003	1	-5.6
ZINC000001649219	1	-5.6
ZINC000100062394	1	-5.6
ZINC000040380449	1	-5.6
ZINC000299817554	1	-5.6
ZINC000085566206	1	-5.6
ZINC000031164529	1	-5.6
ZINC000217634649	1	-5.6
ZINC000004027278	1	-5.6

ZINC000003882001	1	-5.6
ZINC000253390032	1	-5.6
ZINC000103899095	1	-5.6
ZINC000015147904	1	-5.6
ZINC000004521655	1	-5.6
ZINC000005410561	1	-5.6
ZINC000253506258	1	-5.6
ZINC000004096337	1	-5.6
ZINC000248240824	1	-5.6
ZINC000253500805	1	-5.6
ZINC000015209455	1	-5.6
ZINC000253389364	1	-5.6
ZINC000004096262	1	-5.6
ZINC000008762125	1	-5.6
ZINC000299817781	1	-5.6
ZINC000012890604	1	-5.6
ZINC000252505021	1	-5.6
ZINC000000393724	1	-5.6
ZINC000031157798	1	-5.5
ZINC000253390031	1	-5.5
ZINC000095680316	1	-5.5

ZINC000096023698	1	-5.5
ZINC000077268795	1	-5.5
ZINC000004271945	1	-5.5
ZINC000006665108	1	-5.5
ZINC000242437515	1	-5.5
ZINC000015266510	1	-5.5
ZINC000253529985	1	-5.5
ZINC000100779031	1	-5.5
ZINC000005161043	1	-5.5
ZINC000014918740	1	-5.5
ZINC000028536309	1	-5.5
ZINC000028968107	1	-5.5
ZINC000252516284	1	-5.5
ZINC000000062002	1	-5.5
ZINC000000403038	1	-5.5
ZINC000253389368	1	-5.5
ZINC000000057585	1	-5.5
ZINC000013660067	1	-5.5
ZINC000248341254	1	-5.5
ZINC000229772308	1	-5.5
ZINC000248008256	1	-5.5

ZINC000253498760	1	-5.5
ZINC000000391177	1	-5.5
ZINC000017111148	1	-5.4
ZINC000031165820	1	-5.4
ZINC000004083895	1	-5.4
ZINC000028536312	1	-5.4
ZINC000096023885	1	-5.4
ZINC000077269642	1	-5.4
ZINC000248105726	1	-5.4
ZINC000003882002	1	-5.4
ZINC000253387690	1	-5.4
ZINC000000057733	1	-5.4
ZINC000002390999	1	-5.4
ZINC000033495916	1	-5.4
ZINC000031157793	1	-5.4
ZINC000018272247	1	-5.4
ZINC000015205786	1	-5.4
ZINC000253497797	1	-5.4
ZINC000026832404	1	-5.4
ZINC000043763190	1	-5.4
ZINC000013660064	1	-5.4

ZINC000003791929	1	-5.4
ZINC000001041055	1	-5.4
ZINC000000480807	1	-5.4
ZINC000004082037	1	-5.4
ZINC000217634484	1	-5.4
ZINC000100007513	1	-5.3
ZINC000034955329	1	-5.3
ZINC000002556216	1	-5.3
ZINC000238753983	1	-5.3
ZINC000006624114	1	-5.3
ZINC000013533701	1	-5.3
ZINC000043758019	1	-5.3
ZINC000253501835	1	-5.3
ZINC000003951739	1	-5.3
ZINC000038292562	1	-5.3
ZINC000028820378	1	-5.3
ZINC000014723938	1	-5.3
ZINC000022061424	1	-5.3
ZINC000034045609	1	-5.3
ZINC000000136493	1	-5.3
ZINC000031155929	1	-5.3

ZINC000013545047	1	-5.2
ZINC000014588522	1	-5.2
ZINC000003978134	1	-5.2
ZINC000000384010	1	-5.2
ZINC000018256158	1	-5.2
ZINC000299817670	1	-5.2
ZINC000253531476	1	-5.2
ZINC000038611995	1	-5.2
ZINC000015120190	1	-5.2
ZINC000239073255	1	-5.2
ZINC000049888788	1	-5.2
ZINC000085210712	1	-5.2
ZINC000003120705	1	-5.2
ZINC000009007546	1	-5.2
ZINC000014723934	1	-5.2
ZINC000000967176	1	-5.2
ZINC000008580042	1	-5.2
ZINC000001615344	1	-5.2
ZINC000004261954	1	-5.1
ZINC000000518123	1	-5.1
ZINC000005839381	1	-5.1

ZINC000019701862	1	-5.1
ZINC000002562358	1	-5.1
ZINC000004097574	1	-5.1
ZINC000002390750	1	-5.1
ZINC000031155308	1	-5.1
ZINC000000391121	1	-5.1
ZINC000253531475	1	-5.1
ZINC000168509414	1	-5.1
ZINC000014514479	1	-5.1
ZINC000097994617	1	-5.1
ZINC000102818320	1	-5.1
ZINC000038343303	1	-5.1
ZINC000015266506	1	-5.1
ZINC000053715299	1	-5.1
ZINC000005734003	1	-5
ZINC000238743929	1	-5
ZINC000253413458	1	-5
ZINC000085210711	1	-5
ZINC000054956948	1	-5
ZINC000238739052	1	-5
ZINC000043760965	1	-5

ZINC000014723940	1	-5
ZINC000005966484	1	-5
ZINC000032162511	1	-5
ZINC000001529532	1	-5
ZINC000002558641	1	-5
ZINC000001041057	1	-5
ZINC000003897348	1	-5
ZINC000205454665	1	-5
ZINC000031157788	1	-5
ZINC000022065922	1	-5
ZINC000100024490	1	-5
ZINC000001041056	1	-5
ZINC000004666928	1	-5
ZINC000003995890	1	-4.9
ZINC000003861537	1	-4.9
ZINC000003870515	1	-4.9
ZINC000002141018	1	-4.9
ZINC000013612564	1	-4.9
ZINC000004262096	1	-4.9
ZINC000001041054	1	-4.8
ZINC000000391118	1	-4.8

ZINC000001597124	1	-4.8
ZINC000001529819	1	-4.8
ZINC000032302707	1	-4.8
ZINC000005699387	1	-4.8
ZINC000101344509	1	-4.8
ZINC000013527344	1	-4.8
ZINC000002013555	1	-4.7
ZINC000002522771	1	-4.7
ZINC000005699435	1	-4.7
ZINC000003794714	1	-4.7
ZINC000001529820	1	-4.7
ZINC000001555241	1	-4.7
ZINC000038804074	1	-4.6
ZINC000002166531	1	-4.6
ZINC000016698018	1	-4.6
ZINC000004557438	1	-4.6
ZINC000101604212	1	-4.6
ZINC000083255401	1	-4.6
ZINC000002166533	1	-4.6
ZINC000004557439	1	-4.6
ZINC000044075917	1	-4.6

ZINC000001564966	1	-4.5
ZINC000248240826	1	-4.5
ZINC000000396074	1	-4.5
ZINC000001532722	1	-4.5
ZINC000001699441	1	-4.5
ZINC000101724920	1	-4.5
ZINC000000388168	1	-4.4
ZINC000001532712	1	-4.4
ZINC000001677289	1	-4.4
ZINC000038429172	1	-4.4
ZINC000083255403	1	-4.4
ZINC000002166530	1	-4.3
ZINC000248105731	1	-4.2
ZINC000001648294	1	-4.2
ZINC000006845904	1	-4.2
ZINC000001655433	1	-4.2
ZINC000098214460	1	-4.1
ZINC000001672860	1	-4.1

Table S2: Calculated net Binding free energy and energy components values for DosR protein complexes with natural compounds snapshots collected from MD simulation trajectories.

Energies Component (kcal/mol)	M1	M2	M3	M4	reference compound (CID_64945 ursolic acid)
**ΔG Bind**	-38.47	-41.24	-53.51	-32.59	-44.51
**ΔG Bind Coulomb**	-113.27	-97.31	-10.97	-8.92	-24.62
**ΔG Bind Covalent**	-0.19	3.95	3.30	0.42	0.42
**ΔG Bind Hbond**	-1.86	-1.93	-0.59	-1.21	-1.33
**ΔG Bind Lipo**	-4.21	-9.31	-13.76	-8.21	-14.68
**ΔG Bind Packing**	-4.42	-1.87	-3.25	0.00	0.00
**ΔG Bind Solv GB**	127.46	96.58	17.28	15.51	31.43
**ΔG Bind vdW**	-41.98	-51.35	-45.51	-30.18	-35.72
**Lig Strain Energy**	6.08	3.81	2.76	0.93	1.95
